# Upcycled foods: A nudge toward nutrition

**DOI:** 10.3389/fnut.2022.1071829

**Published:** 2022-11-21

**Authors:** Margaret Thorsen, Sheila Skeaff, Francesca Goodman-Smith, Brian Thong, Phil Bremer, Miranda Mirosa

**Affiliations:** ^1^Department of Food Science, University of Otago, Dunedin, New Zealand; ^2^Department of Human Nutrition, University of Otago, Dunedin, New Zealand; ^3^Fight Food Waste Cooperative Research Centre, The University of Adelaide, Urrbrae, SA, Australia

**Keywords:** upcycled food, by-product, nutrition, healthy diet, discretionary food, ultra-processed food

## Abstract

One of the aims of the United Nations Sustainable Development Goals (SDG) is to end hunger and ensure access by all people to safe, nutritious, and sufficient food all year round. An obvious synergy exists between the second SDG “Zero Hunger” and SDG target 12.3 which focuses on halving food waste and reducing food losses. In addition to helping improve global food security, reducing food waste provides financial and environmental benefits. Upcycling food is a technical solution for food waste reduction that retains the nutritional and financial value of food by-products. However, many of the upcycled foods produced are discretionary foods such as biscuits, crackers, and other snack food that are not part of a healthy dietary pattern, and should only be eaten sometimes in small amounts. Given the importance of ensuring a sustainable healthy diet, this paper discusses opportunities for upcycled food manufacturers to produce more nutritious products.

## Introduction

Food is essential not only for our physical wellbeing but food also imparts psychological, social, and cultural benefits. However, our food system increasingly puts pressure on the environment to cope with the demands of a growing global population. Many low- to middle-income countries now have to manage the double burden of malnutrition, whereby undernutrition and obesity co-exist (1). In 2021, 2.3 billion people, or nearly 30 percent of the global population, were moderately or severely food insecure, meaning they did not have access to adequate food (2). Conversely, adult obesity affected 13.1 percent of the global population. Being overweight and obese has been attributed to an increasing proportion of global deaths and disability-adjusted life years due to non-communicable diseases, such as heart disease, diabetes, stroke, and chronic kidney disease (3). Now more than ever, there is a need for healthy and sustainable diets.

Historically, food and nutrition guidelines have provided recommendations for healthy diets with little consideration for sustainability. In 2019, the Eat-Lancet Commission proposed a “planetary” diet good for both human health and environmental sustainability (4). The diet consists primarily of plant foods, including wholegrains, vegetables, legumes, nuts, and seeds, with a relatively small proportion of animal foods (4). In 2021, the Food and Agriculture Organisation of the United Nations (FAO) and the World Health Organisation (WHO) published guiding principles for sustainable, healthy diets (5). The guiding principles promote a wide variety of wholegrains, legumes, nuts, and an abundance and variety of fruit and vegetables, with moderate amounts of eggs, dairy, poultry, and fish, and small amounts of red meat, while restricting highly processed food and drink products.

The United Nations Sustainable Development Goals (SDG) provide a global blueprint to achieve a better and more sustainable future (6). The second SDG, Zero Hunger, aims to end hunger and ensure access by all people to safe, nutritious, and sufficient food all year round by 2030 (7). Given the interconnected nature of the SDGs, achieving Zero Hunger requires progress across multiple other goals and targets. An obvious synergy exists with SDG target 12.3 which focuses on halving food waste and reducing food losses (8, 9). Although the definitions of food loss and waste (often abbreviated as FLW) may vary between entities (10), the Food and Agriculture Organization of the United Nations (FAO) defines food loss as the decrease in edible food mass at the production, post-harvest, and processing stages of the food chain (11). Food waste refers to edible foods discarded at the retail and consumer levels. For the purposes of this article, the term food waste is used to encompass both food loss and waste. In addition to helping improve global food security, food waste reduction provides financial and environmental benefits. The environmental benefits of food waste reduction are well documented and include reduced energy use, food-related greenhouse gas production, water use, land use, and eutrophication (12). Despite the reported advantages, there is continued debate about where to focus efforts to reduce food waste.

Read et al. argued the best environmental gains could be achieved by reducing food waste at the downstream stages of the food supply chain, especially in food processing, food service, and households (12). Reducing food waste in the downstream stages of the food supply chain could potentially lead to reduced input demand from upstream stages, such as primary production and processing (12). Rao et al. supported a focus on reducing food processing waste, which is less dispersed and more homogenous than at other stages of the food supply chain, and making food waste valorization more attractive to the food industry (13). Applying a circular economy model to the food processing sector would ensure better use of food waste, surplus, and by-products (something produced during the manufacture or processing of another product) (14). Alternatively, Augustin et al. have advocated for a focus on reducing fruit and vegetable waste (15) because of the role fruit and vegetable intake plays in disease prevention (16) and data that suggest fruit and vegetables are significant contributors to food loss and waste throughout the food supply chain (17, 18).

The emergence of a fourth industrial revolution (Industry 4.0) presents opportunities to reduce food waste with innovative technologies such as the Internet of Things, smart sensors, and 3D printer technology (19–22). Specifically, upcycling or valorizing food has been proposed as a technological solution to food waste (21) that retains the nutritional and financial value of food by-products (13, 23–25). Upcycled foods are defined as “ingredients that otherwise would not have gone to human consumption, are procured and produced using verifiable supply chains, and have a positive impact on the environment” (26). The extraction of bioactive agents, such as lycopene, beta-carotene, and ferrous sulfate from food by-products may be considered a value-adding or waste-to-value process; the terms value-adding, waste-to-value and upcycling are often used interchangeably within the research literature (27, 28). However, the upcycled food sector aims for whole resource utilization rather than the extraction of nutrients and other bioactive compounds, thus reducing overall food waste (26). The environmental impacts of upcycling food, and the position of upcycled food within the food waste hierarchy, have been the focus of recent debate. Moshtaghian et al. proposed creating a separate level within the food waste hierarchy, specifically for upcycled food (29). Moshtaghian etal. suggest that upcycled food be positioned above animal feed as upcycled food is considered suitable for human consumption, but lower than the redistribution of food, as the production process for upcycled food may have additional negative impacts on the environment.

Upcycled food research to date has typically looked at the nutrients present in the source by-product or food waste and the impact an upcycled ingredient has on the nutrient composition of the end product (23, 30–33). For example, grape pomace was used to increase the antioxidant content and nutritional profile of 3D printed cookies made from broken wheat which would have otherwise been sent for animal feed (19). Grasso et al. suggest the upcycled food industry could gain marketing opportunities by improving the nutrient composition of an end product; for example, when a proportion of wheat flour is replaced with upcycled sunflower flour, muffins have increased levels of insoluble fiber, protein, mineral content, and antioxidants (23). There are conflicting results as to how much consumers value the increased nutrient content versus the environmental benefits of upcycled food. Two studies suggest the increased nutrient content of products containing upcycled ingredients did not create a willingness to pay for environmental-focused consumers (34, 35). Conversely, consumers reported a willingness to pay a premium price when nutritional and/or environmental information about upcycled food was provided (36). Regardless of a consumer’s willingness to pay, upcycled food manufacturers need to be mindful of the nutritional contribution their products make to the range of foods available for purchase. Consumers may be given a false sense of confidence about the nutritional quality of a product, and therefore the overall quality of their diet when upcycled ingredients are added to discretionary or ultra-processed foods (UPF). Given the importance of ensuring a sustainable healthy diet, this paper discusses some of the nutritional opportunities and pitfalls for the upcycled food sector, with the aim to nudge upcycled food manufacturers toward more nutritious products.

## Discussion

Rao et al. proposed a decision tree to determine whether valorizing a food by-product was sustainable, safe, and nutritionally relevant (13). The initial step in this decision tree queries whether there are favorable environmental, economic, and social outcomes from valorizing an identified by-product. The second step in the decision tree determines whether the resulting product will be safe for human consumption. Examples of safety considerations include the potential risk of Prion diseases, toxic molds, and the content of heavy metals (13). The final step considers whether the upcycled food adds value to the human diet. Upcycled food manufacturers can take into consideration several factors when determining the nutritional contribution of an upcycled food to the diet, including the nutritional properties of the source product; the nutritional qualities of the end product; determining whether an end product was an ultra-processed food; and looking for opportunities to improve the nutritional quality of upcycled foods available for purchase.

### Nutritional properties of the source product

Healthy eating guidelines promote the consumption of unprocessed or minimally processed foods (5, 37). For this reason, efforts to direct edible but unmarketable produce, such as “ugly” or misshaped fruit and vegetables, toward human consumption in their current form, or used to create products such as soups, sauces, or chutneys/relishes, would achieve the optimal nutrition outcome for this source of food waste. Redirecting edible, unmarketable produce for human consumption has the lowest environmental impact (29). However, there is a wide array of by-products produced from the food manufacturing processes that have the potential to provide a consistent source of material rich in various nutrients. The upcycled food industry should primarily focus on upcycling by-products that align with the guiding principles for a sustainable healthy diet, including wholegrains, legumes, nuts, vegetables, and fruits (5), and thus ensure upcycled ingredients improve the nutrient quality of the food supply. Conversely, less effort should be directed to upcycling foods and by-products with a poorer nutrient profile such as refined grains, animal fats, pastries, and bakery items with a lower fiber, and higher added sugar, saturated fat, or salt content. For example, salt is an essential ingredient in bread production, but by adding upcycled bread to otherwise salt-free foods, manufacturers are inadvertently increasing the salt or sodium content of the food supply. Kalmpourtzidou et al. suggested that, in 88% of countries, vegetable intake was below the recommended three serves of vegetables per day and that vegetable supply in 61% of countries was insufficient to meet minimum vegetable recommendations (38). Given the evidence supporting fruit and vegetable consumption and the prevention of cardiovascular disease, certain cancers, and depression (3, 39), fruit and vegetable by-products, in particular, should be a priority for upcycling.

Research is underway exploring the nutritional and functional impact of using fruit pomaces as upcycled ingredients in different foods (30, 31, 40–42). Grape pomace, a by-product of wine production, is a rich source of dietary fiber (43, 44), protein (44), and phenolic compounds (43–45). Olive pomace or pâté from olive oil production is also a source of phenolic compounds and oleic acid, a monounsaturated fat (46). Other by-products currently being researched for their nutrient composition and functional contribution to the food supply chain include brewer’s spent grain (47–49) and okara (27, 50).

To date, upcycled food research has typically taken a reductionist approach (i.e., single nutrient approach) when evaluating the value of source products (23, 30, 40, 41). Upcycled food manufacturers need to be mindful of the shift in global health messaging toward recommending increased consumption of whole foods, unprocessed or minimally processed, and minimizing consumption of UPF (5). Unprocessed and minimally processed foods contain numerous nutrients within complex matrices which influence nutrient bioaccessibility and bioavailability (51, 52). Thus, a diet based on whole foods may have a different effect on health indicators when compared to single nutrients assessed in isolation (53). Many food by-products are processed and the matrix of the source food has been modified. Nevertheless, upcycled food manufacturers can aim for full resource utilization, and work to limit further processing to that which enhances food safety and is practical for human consumption, while avoiding the production of UPF where possible.

### Nutritional qualities for end products

Many studies evaluating the end use of upcycled ingredients have focused on creating discretionary foods such as biscuits, crackers, and other snack food (23, 48) with an improved amino acid profile, higher fiber, mineral, and antioxidant content, or with lower glycemic index compared to a standard product. The Australian Dietary Guidelines describe discretionary choices as being “foods and drinks that are not an essential part of healthy dietary patterns, are high in kilojoules, saturated fat, added sugars, and salt or alcohol, and if chosen, should be eaten only sometimes and in small amounts” (37). Furthermore, commercially produced discretionary foods may be classified as UPF and drinks which have been associated with poorer health outcomes (54). The inclusion of an upcycled ingredient may inadvertently create a nutritional halo for discretionary food.

The supply of higher-quality food from upcycled food waste may be achieved if upcycled food manufacturers focus on producing staple foods, including bread, pasta, and noodles, cow’s milk alternatives, and longer shelf-life fruit and vegetables. Okara is an example of a soy-based by-product rich in dietary fiber (55), protein (56), and mono and polyunsaturated fats (57) used to enrich staple foods for the Asian market. Research has shown the glycemic index of rice noodles and steamed rice bread can be lowered, and the prebiotic content increased by adding okara (50, 58). Restrictive diets, such as a gluten-free diet, which are recognized as being lower in fiber, and higher in saturated fat and added sugar (59), would also benefit from the addition of nutrient-rich upcycled ingredients to staple food items. For example, the addition of plant-based food waste and by-products, such as vegetables, fruit, cereals, and legumes have been trialed in gluten-free pasta, with the advantage of increasing the dietary fiber, protein, and micronutrient content of end products (32).

Current food labeling legislation does not specifically state requirements for products containing upcycled ingredients. However, based on Aotearoa New Zealand retail food labeling requirements (60), upcycled food manufacturers will be required to provide a list of all ingredients, and corresponding allergens, in descending order, including those present in any upcycled ingredients. The length of an ingredient list when upcycling manufactured foods, such as bread, and bakery items may be problematic when limited packaging space is available. As the upcycled food industry grows, clarification of best practice when constructing the ingredient list for upcycled foods may be required.

To build consumer confidence, the Upcycled Food Association developed a certification standard for upcycled food products to verify the input ingredients as upcycled and ensure they are present in meaningful amounts in the finished product (61). Currently, the standard does not include nutritional criteria as part of its eligibility assessment; this should be considered in future iterations of the standard.

### A focus on ultra-processed foods

The guiding principles for a sustainable healthy diet specifically mention minimizing the intake of UPF (5). The NOVA classification system was developed as a means to assess the degree of industrial processing a food had undergone (62). Food can be classified into four groups; group 1 (unprocessed and minimally processed foods), group 2 (processed culinary ingredients), group 3 (processed foods), and group 4 (UPF). Ultra-processed foods have undergone the process of fractioning whole foods into their individual substances, such as proteins, fats, sugars, starches, and fiber. Some of these substances may then undergo further industrial processing, such as hydrolysis, hydrogenation, or chemical modifications (62). The final assembly of a UPF involves combining modified and unmodified food substances, and frequently food additives, using industrial techniques such as extrusion, molding, and pre-frying. Sugar, fats, and salt are also frequently added to UPF. The processes and ingredients used to produce UPF can create nutritionally unbalanced foods and consequently have been associated with poorer health outcomes (62). Furthermore, UPFs are linked to negative environmental impacts, such as higher energy use, biodiversity loss, greenhouse gas emissions, and land and water use (63), and more nutritious foods are often more environmentally sustainable (64). The NOVA classification system provides a useful tool for upcycled food manufacturers working to produce healthier end products. By focusing upcycled food production toward groups 1–3 on the NOVA criteria, such as upcycled flours or canned produce, and away from UPFs, upcycled food manufacturers could make a more positive contribution to the food supply chain.

Nutrient-rich by-products such as fruit pomaces or brewer’s spent grain require further industrial processing to ensure safety for human consumption (13). In these situations, upcycled food manufacturers may be unable to avoid producing a UPF. Nevertheless, there may still be nutritional benefits to be gained, when compared to other UPF, if upcycled food manufacturers include wholegrains, nuts, seeds, fruits, and vegetables, and minimize the total energy, added sugar, fat, and salt content in the UPF. Some countries have tools that support food manufacturers to optimize the nutritional content of packaged foods, such as the Health Star Rating (HSR) in Australia and Aotearoa New Zealand (65), and the Nutri-Score in Europe (66). Alternatively, Davidou et al. have proposed the Siga classification as a variation on the NOVA criteria, whereby the UPF category is subdivided into three subcategories, enabling further classification and differentiation of UPFs (67). Nutritionally balanced UPFs (i.e., lower in added sugar, fat, and salt content), with one marker of ultra-processing (MUP), were assessed more favorably than nutritionally unbalanced UPFs with one MUP, or products with more than one MUP (67). The Siga classification could also serve as a tool to nudge upcycled food production toward healthier end products.

## Future research and conclusion

To better understand the nutritional quality of upcycled foods currently available, existing products could be assessed against the HSR, the Nutri-score, and the NOVA criteria. The proposed Siga criteria may provide a more detailed analysis of upcycled foods classified as UPFs, thus providing more specific information about the potential to improve the nutritional quality of the end product. Gaining an understanding of upcycled food manufacturers’ interpretation of a sustainable healthy diet, and their intentions to produce nutritious foods when developing upcycled products will inform nutrition and public health professionals about the support they can provide the upcycled food sector.

**FIGURE 1:**
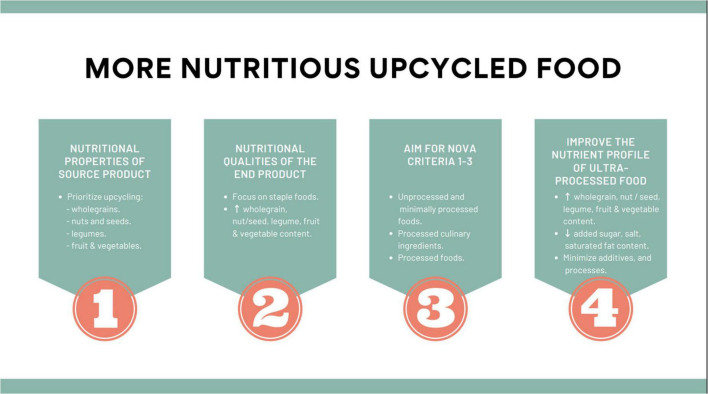
A summary of key factors to creating more nutritious upcycled food.

The inclusion of nutrition criteria in the Upcycled Food Association certification standard may help shift the direction of the upcycled food sector toward more nutritious upcycled foods by nudging manufacturers toward products that more closely align with a sustainable and healthy diet. Targeted nutrition-related questions could also be included in a decision-making tree for upcycled food manufacturers, such as that proposed by Rao et al. (13). Questions could focus the upcycled food manufacturers’ attention toward optimizing nutritional outcomes during the upcycling process, including the alignment of the source product with foods promoted by healthy food guidelines, determining whether the end product would serve as a staple or discretionary food, and where the end product would sit on the NOVA scale. If the end product was classified as a UPF, upcycled food manufacturers could be encouraged to consider how to optimize the nutritional quality of the end product by increasing the content of wholegrains, nuts, seeds, fruits, and vegetables while lowering the total energy, added sugar, fat and salt content. A summary of the key points discussed in the article has been summarized in Figure 1. Upcycling food has been presented as an opportunity to reduce food waste and thus provide positive environmental, societal, and economic outcomes. With careful consideration of the nutritional properties of the source product, and the nutritional qualities of the end product, upcycled foods can also positively contribute to a healthy diet.

## Data availability statement

The original contributions presented in the study are included in the article/supplementary material, further inquiries can be directed to the corresponding author.

## Author contributions

MT, SS, FG-S, PB, and MM: conceptualization. MT and BT: investigation. MT: writing—original draft preparation. SS, FG-S, BT, PB, and MM: writing—review and editing. SS, PB, and MM: project administration. PB and MM: funding acquisition. All authors have read and agreed to the published version of the manuscript.
